# Diverse Physiological Roles of Kynurenine Pathway Metabolites: Updated Implications for Health and Disease

**DOI:** 10.3390/metabo15030210

**Published:** 2025-03-20

**Authors:** Yuechang Wang, Yonggang Zhang, Wei Wang, Yanmin Zhang, Xueqian Dong, Yang Liu

**Affiliations:** Shandong Food Ferment Industry & Design Institute, QiLu University of Technology (Shandong Academy of Sciences), No. 41, Jiefang Road, Jinan 250013, China

**Keywords:** tryptophan, kynurenine pathway, oxidative stress, neurotoxicity, neurodevelopmental disorders

## Abstract

Tryptophan is an essential amino acid critical for human health. It plays a pivotal role in numerous physiological and biochemical processes through its metabolism. The kynurenine (KYN) pathway serves as the principal metabolic route for tryptophan, producing bioactive metabolites, including KYN, quinolinic acid, and 3-hydroxykynurenine. Numerous studies are actively investigating the relationship between tryptophan metabolism and physiological functions. These studies are highlighting the interactions among metabolites that may exert synergistic or antagonistic effects, such as neuroprotective or neurotoxic, and pro-oxidative or antioxidant activities. Minor disruptions in the homeostasis of these metabolites can result in immune dysregulation, contributing to a spectrum of diseases. These diseases include neurological disorders, mental illnesses, cardiovascular conditions, autoimmune diseases, and chronic kidney disease. Therefore, understanding the physiological roles of the KYN pathway metabolites is essential for elucidating the contribution of tryptophan metabolism to health regulation. The present review emphasizes the physiological roles of KYN pathway metabolites and their mechanisms in disease development, aiming to establish a theoretical basis for leveraging dietary nutrients to enhance human health.

## 1. Introduction

Tryptophan, also referred to as β-indolylalanine, is the sole essential aromatic amino acid characterized by an indole structure [1]. Among the 20 natural amino acids, tryptophan has the highest molecular weight. However, it is the least abundant in proteins and cells, serving as a biosynthetic precursor for numerous microbial and host metabolites [1]. Dietary intake is the principal method for the human body to obtain tryptophan. The World Health Organization recommends a daily tryptophan intake of 3.5–6.0 mg/kg body weight for adults [2,3]. Notably, no adverse effects of excessive dietary tryptophan intake have been reported to date [4]. 

Under normal physiological conditions, less than 1% of tryptophan is utilized for protein synthesis. The majority of tryptophan enters the gastrointestinal tract, where a small fraction is metabolized, while the remainder enters the portal circulation for hepatic metabolism [5,6]. Tryptophan performs various biological functions, including regulating immune responses [7], maintaining gut microbiota homeostasis [8], participating in redox reactions [9], modulating metabolism [10], and regulating neuronal functions [11]. Insufficient tryptophan intake can result in several diseases, including pellagra [12], Kawasaki disease [13], depression [11], and inflammatory bowel disease [14]. 

Numerous studies have established a close relationship between tryptophan metabolism and related physiological and pathological processes. The kynurenine (KYN) pathway is one of the most extensively studied tryptophan metabolic pathways. Advancing our understanding of the functions of metabolites in the tryptophan–KYN pathway and elucidating the mechanisms underlying their physiological roles are essential for investigating the bioactivity of tryptophan and its impact on human health. The present review delves into the physiological functions of metabolites in the KYN pathway and their roles in health and disease. This review paves the way for future research on tryptophan functionality, related disease treatments, and the development of targeted drugs. 

## 2. Tryptophan–Kynurenine Pathway and Its Metabolites

**Figure 1:**
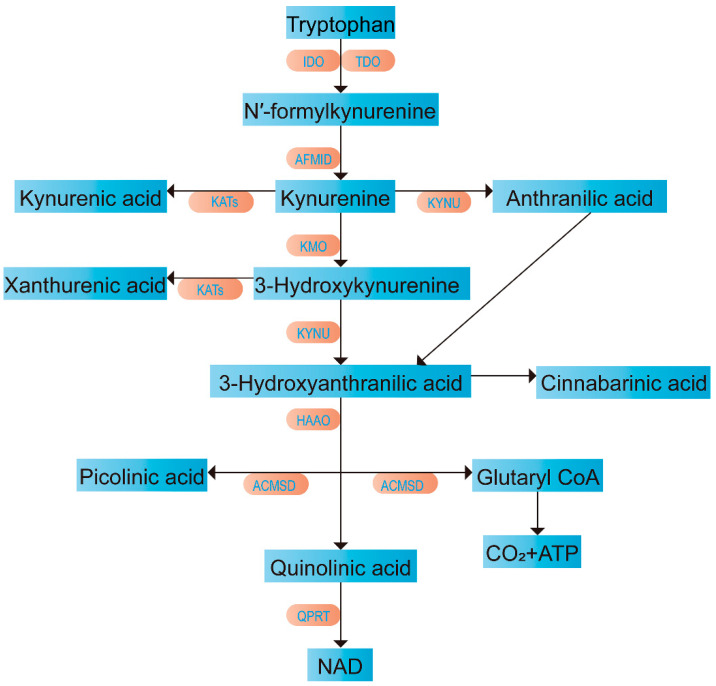
The kynurenine pathway. IDO, indoleamine 2,3-dioxygenase; TDO, tryptophan 2,3 dioxygenase; AFMID, kynurenine formamidase; KMO, kynurenine 3-monooxygenase; KATs, kynurenine aminotransferases; KYNU, kynureninase; HAAO, 3-hydroxyanthranilate 3,4-dioxygenase; ACMSD, α-amino-β-carboxymuconate-ϵ-semialdehyde decarboxylase; QPRT, quinolinate phosphoribosyltransferase.

Under normal physiological conditions, tryptophan is metabolized as a free amino acid [15]. It promotes cell growth and plays a crucial role in physiological processes, such as biochemical regulation and signal transduction [16]. The metabolic pathways of tryptophan include the KYN, the 5-hydroxytryptamine (5-HT), and microbial metabolic pathways [17]. As mentioned previously, the KYN pathway is the primary metabolic route for tryptophan. Specifically, it accounts for over 90% of tryptophan metabolism and leads to the production of several bioactive metabolites. Figure 1 illustrates the complete KYN pathway process.

Tryptophan is initially metabolized into *N*′-formylkynurenine (NFK) through the catalysis of indoleamine 2,3-dioxygenase (IDO) or tryptophan 2,3-dioxygenase (TDO) and subsequently hydrolyzed to KYN. In the microglial cells of the brain, KYN is converted into 3-hydroxykynurenine (3-HK), which is further metabolized into 3-hydroxyanthranilic acid (3-HAA). Subsequently, under the catalysis of 3-hydroxyanthranilate 3,4-dioxygenase (HAAO), 2-amino-3-carboxymuconate semialdehyde (ACMS) is produced in brain microglia and chemotactic macrophages. ACMS spontaneously rearranges into quinolinic acid (QA) within these cells [18]. QA is then further metabolized into nicotinamide riboside (NR), which is ultimately converted into nicotinamide adenine dinucleotide (NAD) [19], facilitating energy production.

The KYN pathway involves several additional metabolic branches. Under the catalysis of kynureninase (KYNU), KYN can bypass 3-HK. It is initially metabolized into anthranilic acid (AA) and subsequently converted into 3-HAA [20]. 3-HAA can be converted into picolinic acid (PA) within glial cells or neurons [21], or it can undergo auto-oxidation to form cinnabarinic acid (CA) [22]. KYN can also be metabolized into xanthurenic acid (XA) through 3-HK. In astrocytes within the brain, KYN can be metabolized into kynurenic acid (KYNA) [23]. Another enzyme, nicotinate phosphoribosyltransferase domain-containing 1 (NAPRT1), catalyzes the conversion of NR into nicotinic acid (NA) [1]. A detailed overview of the metabolites involved in the KYN pathway is provided in Table 1.

## 3. Physiological Functions of Kynurenine Metabolites and Their Impact on Health and Disease

Increasing evidence suggests that tryptophan metabolism directly influences various physiological functions of the host and is significantly correlated with the homeostasis of KYN pathway metabolites. The metabolites in the KYN pathway exhibit either cooperative or opposing actions. These include neuroprotective or neurotoxic effects, and pro-oxidative or antioxidative activities. Under normal physiological conditions, the KYN pathway maintains homeostasis, properly regulating physiological functions. However, under abnormal conditions, such as environmental stress and inflammation, the metabolic state and levels of KYN pathway metabolites change, resulting in homeostatic imbalance. This imbalance is implicated in the development of various diseases, including neurological disorders, psychiatric disorders, cardiovascular diseases, autoimmune diseases, chronic kidney disease, neoplasms, and diabetes [91,92,93,94,95,96,97].

### 3.1. Quinolinic Acid (QA)

QA, characterized by its pyridine ring structure, is a downstream metabolite of the KYN pathway. It is primarily produced in activated microglia and macrophages, where it functions as a neurotransmitter.

**Inflammatory promotion** Typically, QA cannot cross the blood–brain barrier. However, under abnormal conditions, microglia within the brain synthesize QA, resulting in elevated QA levels in the brain. External environmental disturbances lead to increased expression of interferon-gamma (IFN-γ) [98], subsequently enhancing the activity of IDO, KYNU, and kynurenine 3-monooxygenase (KMO) [42]. During this period, the ability of macrophages to synthesize QA is enhanced to 20–30 times that of microglia. At the same time, the expression of enzymes related to the QA metabolic process is inhibited [42]. This increased metabolic activity within the tryptophan-QA pathway [97] and the obstruction of QA metabolism lead to an excessive accumulation of QA. Excessive levels of QA activate N-methyl-D-aspartate (NMDA) receptors and inhibit the reuptake of glutamate by astrocytes. QA accumulation upregulates the expression of chemokines, including stromal cell-derived factor 1α, chemokine CXC ligand 9, and chemokine ligand 1. It also increases the expression of chemokine receptors, such as chemokine receptor 4, CC chemokine receptor 5, and CC chemokine receptor 3, in astrocytes. This process promotes brain inflammation [99] and may induce other disease-related physiological imbalances. These imbalances are key mechanisms in various neurological and psychiatric disorders. Notably, the brains of patients with depression reportedly exhibit increased macrophage activity, enhanced QA metabolic activity, and activation of NMDA receptors [17]. Consequently, NMDA receptor blockers significantly alleviate depression symptoms by inhibiting QA signaling and preventing Ca^2+^ influx into cells [43,44,45,100]. Moreover, regarding KYN pathway metabolites, QA serves as a crucial precursor for NAD synthesis. Current treatments aim to limit QA synthesis while increasing KYNA production. However, it is essential to consider the impact on NAD synthesis to prevent NAD deficiency.

**Inducing oxidative stress** The imbalanced expression of QA can result in severe oxidative stress symptoms. For example, QA overexpression leads to the formation of complexes with metal ions, generating substantial quantities of free radicals, reactive oxygen species (ROS), and lipid peroxides [35]. This subsequently impacts glutathione levels and the activity of superoxide dismutase dependent on copper (Cu) and zinc (Zn) [70,101]. Moreover, QA can induce oxidative damage without activating NMDA receptors. Instead, it forms QA-Fe^2+^ complexes with Fe^2+^, which enhance hydroxyl radical formation via the Fenton reaction. This process plays a crucial role in hydroxyl radical-mediated DNA strand breaks and may contribute to neuronal apoptosis [44,102,103,104]. Furthermore, QA enhances the neurotoxicity of 3-HK, exhibiting a synergistic effect on ROS production [102,105]. Oxidative stress induced by QA increases the permeability of the blood–brain barrier, facilitating enhanced crosstalk between peripheral tissues and the brain. Consequently, macrophage infiltration from peripheral tissues into the brain increases, damaging the brain tissue structure and neuronal function [61], and leading to apoptosis of neurons and astrocytes [106]. This physiological response occurs extensively in regions such as the hippocampus, striatum, and neocortex [35]. It also occurs in the areas involved in learning and memory, rendering them more sensitive to the neurotoxic effects of QA [46,47,107]. Therefore, abnormal accumulation of QA can significantly impact cognitive, memory, and learning functions.

**Inhibition of inflammation** In a rat model of colitis, treatment with QA can alleviate the symptoms of ulcerative colitis. The mechanism involves QA inhibiting the Toll-like receptor 4/nuclear factor κB (NF-κB) and NF-κB/inducible nitric oxide synthase/nitric oxide signaling pathways. Additionally, QA reduces intestinal inflammatory responses and mucosal damage by decreasing the expression of pro-inflammatory cytokines, such as interleukin (IL)-6, IL-1β, and tumor necrosis factor-alpha (TNF-α) [108]. In human colon cancer cells (LS180 cells), QA can regulate the expression of the tumor suppressor gene *p53* in a concentration- and time-dependent manner, promoting its phosphorylation. This mechanism effectively inhibits the proliferation of LS180 cells [50], with the effect peaking at a QA concentration of 2.5 mM. Moreover, QA effectively inhibits the proliferation of colon cancer cells in vitro, indicating a positive regulatory effect. This effect primarily occurs through the modulation of several signaling pathways and protein expressions, including the extracellular signal-regulated kinase, p38 mitogen-activated protein kinase, and phosphatidylinositol 3-kinase/protein kinase B pathways [49,50]. Overall, these findings suggest that QA possesses anti-inflammatory functions under certain physiological conditions, primarily targeting peripheral organs. However, its mechanism of action requires further investigation.

**Figure 2:**
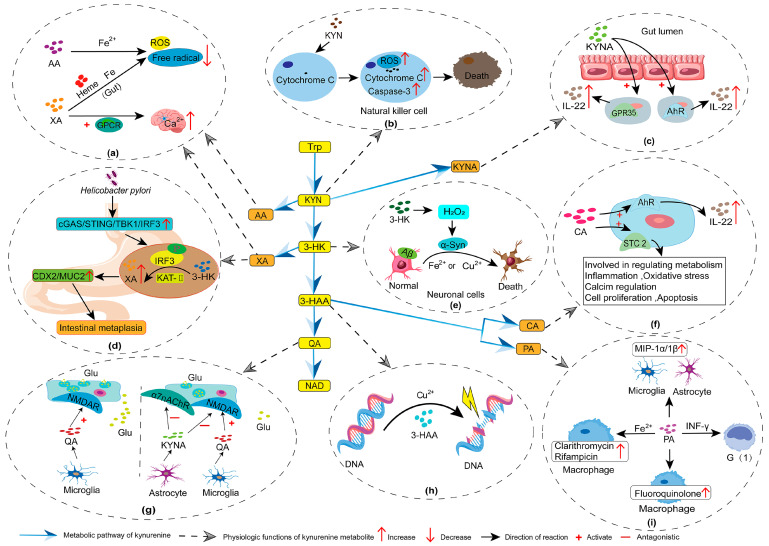
The physiological effects of KYN pathway metabolites. (**a**) AA can form complexes with Fe^2+^, downregulating ROS levels, and exhibiting antioxidant activity; XA exerts antioxidant effects by binding to heme and iron (Fe) chelators; XA facilitates neural signal transmission in the brain by activating cation channels mediated by GPCR and increasing intra-cellular Ca^2+^ concentrations. (**b**) In NK cells, KYN promotes the increase in cytochrome C and caspase-3, inducing elevated free radical levels, and leading to cell death. (**c**) In intestinal epithelial cells, KYNA activates GPR35 and AhR receptors, promoting increased IL-22 levels. (**d**) In the stomach, helicobacter pylori activates the cGAS/STING/TBK1/IRF3 signaling pathway, promoting XA production, and leading to gastric metaplasia. (**e**) 3-HK auto-oxidizes to produce H_2_O_2_, which is further derived into α-Syn, binding to Aβ and complexing with metal ions, causing cell death. (**f**) CA activates AhR receptors and STC2 signaling, providing a protective effect on cells. (**g**) In microglia, QA activates NMDA receptors and promotes Glu release. In astrocytes, KYNA acts as an antagonist of α7nAChRs and NMDA receptors, thereby mitigating the effects of QA. (**h**) 3-HAA disrupts DNA structure by chelating metal ions. (**i**) Under INF-γ stimulation, PA can control cells in the G1 phase; PA activates microglia and astrocytes, promoting increased MIP-1α/1β levels, exhibiting antiviral effects; PA enhances the action of antibacterial drugs in macrophages by chelating metal ions. KYN, kynurenine; KYNA, kynurenic acid; AA, anthranilic acid; 3-HK, 3-hydroxykynurenine; XA, xanthurenic acid; 3-HAA, 3-hydroxyanthranilic acid; CA, cinnabarinic acid; PA, picolinic acid; QA, quinolinic acid.

**Affecting neural signal transmission** Elevated levels of QA can disrupt the glutamate transport system, affecting both the uptake and release of glutamate [109]. This disruption further inhibits glutamate reuptake by astrocytes and suppresses the expression of glutamine synthetase. Consequently, ionotropic glutamate receptors are activated, further disrupting the dynamic balance of the glutamate–glutamine-GABA cycle (Figure 2g). Additionally, the low capacity of QA for glutamate uptake results in its prolonged presence in the synaptic cleft. This prolonged presence enhances neurotoxicity and neuronal damage, contributing to mood disorders [110]. However, QA can interact with Fe^2+^ or Cu^2+^ to form coordination complexes, promoting ROS generation. This interaction increases glutamate release, leading to lipid peroxidation and DNA damage [44,103]. Therefore, the impact of QA on neural signal transmission stems from its interaction with various biochemical substances.

### 3.2. Kynurenine (KYN)

KYN is the primary metabolite in the tryptophan–KYN pathway. Excessive consumption or supplementation of tryptophan can directly influence the metabolism of KYN. This, in turn, alters the balance of its downstream metabolites and influences both the 5-HT metabolic pathway and the microbial metabolic pathway of tryptophan [111].

**Neurotoxicity** Various neurological and psychiatric disorders, including schizophrenia spectrum disorders, are associated with abnormal KYN levels [112]. For example, KYN induces neurotoxicity through two primary mechanisms. In the IDO-dependent metabolism, inflammatory responses upregulate pro-inflammatory cytokines mediated by T lymphocytes and macrophages, including INF-γ and INF-β [113]. These cytokines activate IDO expression, promoting KYN synthesis and increasing the KYN/tryptophan ratio [113,114]. In IDO-independent metabolic pathways, excessive IFN-α may induce the tryptophan–KYN pathway during inflammatory conditions, leading to KYN accumulation [61,115]. KYN acts as an agonist of glutamate receptors. Elevated KYN concentrations result in the overexpression of these receptors, leading to neuroexcitation and cellular Ca^2+^ overload. These factors are critical in cerebral ischemia and are associated with the onset of numerous neurological disorders [27]. Additionally, KYN induces the release of cytochrome C and the activation of caspase-3. It also inhibits the expression of natural killer (NK) cell-activating receptors, such as NKG2D, and natural cytotoxicity receptors, including NKp46, thereby suppressing NK cell growth and impairing their function [116]. This process involves extensive consumption of antioxidants and the generation of large quantities of free radicals, ultimately contributing to neurotoxicity [28] (Figure 2b). At the same time, immune cells require large amounts of NAD to manage inflammatory responses, which leads to increased metabolism through the KYN-QA-NAD pathway. Therefore, the neurotoxicity of excessive KYN may also be associated with QA.

**Antioxidant activity** Metabolic homeostasis of KYN is crucial for the nervous system. Regulating its disrupted metabolic process can enhance the physiological state of the nervous system. A typical example is that exercise effectively alleviates stress in mice and prevents stress-induced depressive symptoms. This effect is primarily mediated through the upregulation of kynurenine aminotransferases (KATs), which enhances metabolic KYN consumption [1]. In the absence of metabolic overload, KYN can cross the blood–brain barrier and increase the synthesis of the KYN-KYNA pathway without overburdening the QA metabolic pathway. This helps maintain a balance between neurotoxicity and neuroprotection [27,117] (refer to Section 3.5 for the specific mechanism of KYNA). In rat brain tissue, the administration of 0.1 µM KYN increases the levels of glutathione, glutathione reductase, and glutathione peroxidase [117]. This process involves the reaction of KYN with hydroxyl radicals and peroxynitrite to form KYNA [30,118]. Additionally, KYN can decrease the production of radicals and lipid peroxidation induced by pro-oxidants, such as ferrous sulfate, peroxynitrite, and 3-nitropropionic acid. This helps prevent the oxidative degradation of DNA and proteins, thereby protecting the nervous system [117,119,120]. However, the radical-scavenging effect of KYN is selective. Although KYN is an effective scavenger of hydroxyl radicals and peroxynitrite radicals, it is ineffective against superoxide anion radicals and hydrogen peroxide [117].

### 3.3. 3-Hydroxykynurenine (3-HK)

3-HK is a metabolite derived from KYN that can cross the blood–brain barrier from peripheral tissues. It can be synthesized in astrocytes and microglia within the brain [121].

**Oxidative stress** 3-HK primarily exhibits neurotoxicity, with its neurotoxic effects in the brain being over four times greater than those of QA [33]. At considerably low concentrations (1 µM), 3-HK exerts potent neurotoxic effects on brain neurons [33]. This indicates that 3-HK plays a dominant role in mediating the neurotoxic effects of the KYN pathway. However, multiple factors influence the intensity of 3-HK neurotoxicity. Among these factors, the most critical is the activity of amino acid transporters involved in the cellular uptake of 3-HK. Competitive interactions limit the cellular uptake of 3-HK, resulting in either small amounts or no uptake at all, thereby reducing its neurotoxic effects. In regions such as the cortex and striatum, amino acid transporters exhibit higher activity than those in the cerebellum, leading to heightened neurotoxicity of 3-HK in these regions. In contrast, the cerebellum demonstrates the lowest level of 3-HK uptake, resulting in reduced neurotoxicity [33].

The primary mechanism by which 3-HK causes toxicity is by inducing oxidative stress. Upon entering the cell, 3-HK interacts with cellular xanthine oxidase, resulting in the significant production of ROS. This, in turn, induces internucleosomal DNA damage and leads to apoptosis [39]. Notably, 3-HK can also generate self-oxidation products, such as hydrogen peroxide, through auto-oxidation [102]. These auto-oxidation products bind with specific amino acid residues—lysine and histidine—in α-synuclein and amyloid-β (Aβ) peptides. This binding increases protein modifications in the presence of Cu^2+^ or Fe^3+^, enhancing the oxidative environment in cells and leading to cellular damage [122] (Figure 2e). However, this effect is dose- or target-dependent. For example, at concentrations below 100 µM, 3-HK exhibits antioxidant properties temporarily, although these effects diminish over time. This antioxidant function is speculated to originate from auto-oxidation. At concentrations exceeding 100 µM, 3-HK exhibits pro-oxidative properties [34]. Glial cell lines, such as C6 glioma cells, or cortical homogenates containing glial cells are widely used in studies investigating the relationship between 3-HK and oxidative stress. However, these systems contain antioxidant enzymes, such as glutathione and catalase, making it difficult to determine the precise source of antioxidant activity [34]. Thus, the relationship between 3-HK and cellular oxidative stress requires further investigation. Notably, the interaction between 3-HK and QA enhances the neurotoxicity of both components. This promotes the degeneration and apoptosis of neuronal cells in brain tissue, as well as free radical production and oxidative stress [123].

**Cataract induction** In addition to inducing oxidative stress, 3-HK is well-known for its role in filtering ultraviolet light in the eye lens. 3-HK and its metabolites are contributing factors to the development of senile cataracts. Notably, elevated activity of KATs is observed in the lenses of older individuals. The primary mechanism involves the conversion of 3-HK into XA under the catalysis of KATs. XA exists in equilibrium with its tautomeric form, 8-hydroxyxanthurenic acid, before being oxidized to form 3-hydroxyanthranilic acid. This fluorescent compound can accumulate in the lens, form conjugates with glutathione, and interact with lens proteins, thereby contributing to cataract formation [31,32].

### 3.4. 3-Hydroxyanthranilic Acid (3-HAA)

3-HAA is a metabolite of the KYN pathway, generated either through the metabolic conversion of 3-HK or the oxidation of AA [124].

**Antioxidant activity** Owing to its ability to dimerize with free radicals, 3-HAA demonstrates significant antioxidant and radical-scavenging activities [36,37]. Under immune imbalance conditions, IFN-γ acts as an inducer to promote the production of 3-HAA. This helps prevent the further depletion of α-tocopherol in lipoproteins and the accumulation of lipid hydroperoxides [125], reflecting its antioxidant function. However, in macrophages, excessive levels of 3-HAA may instead promote apoptosis, as they increase the levels of catalase, superoxide dismutase, and Mn^2+^ [126]. As 3-HAA is a precursor of QA, this apoptosis-inducing mechanism is likely related to the neurotoxic effects of QA [127].

**Anti-inflammatory activity** In addition to its antioxidant properties, 3-HAA also demonstrates anti-inflammatory effects. In microglia and astrocytes, 3-HAA serves as an inducer that can suppress the expression of pro-inflammatory cytokines and chemokines. It can also mitigate cytokine-induced neuronal cell death and confer anti-inflammatory and neuroprotective effects by inducing the expression of heme oxygenase-1 [38]. Furthermore, 3-HAA can downregulate the phosphorylation of stress-activated protein kinases and p38 mitogen-activated protein kinases. 3-HAA also inhibits the activation of dendritic cells and T lymphocyte responses and decreases the production of pro-inflammatory cytokines, including IL-12, IL-6, and TNF-α [128].

**Genotoxicity** The genotoxicity of 3-HAA manifests primarily through its synergistic interaction with metal cofactors such as Cu^2+^. This toxic effect originates from the interaction of the ortho-hydroxyl group of 3-HAA with Cu^2+^. In the presence of Cu^2+^, 3-HAA induces DNA strand breaks in a dose-dependent manner, causes nicks in plasmids, and results in plasmid relaxation [39], contributing to genotoxicity (Figure 2h).

### 3.5. Kynurenic Acid (KYNA)

KYNA is a significant neuromodulator, predominantly synthesized and released by astrocytes. It exerts its regulatory effects by interacting with glutamate receptors and other receptor types.

**Neuroprotection** KYNA is generally regarded as a neuroprotective agent [51]. Its excessive activation can result in an overload of metal ions, such as Ca^2+^, within cells, leading to cellular dysfunction and neurotoxicity. As an antagonist of NMDA receptors, KYNA can mitigate the effects of metabolites such as QA [27,124] (Figure 2g). Consequently, KYNA plays a crucial role in maintaining the function of the nervous system. Additionally, KYNA can inhibit the expression of α 7 nicotinic acetylcholine receptors (α7nAChRs) (Figure 2g), AMPA receptors, and kainate receptors. This action subsequently reduces glutamatergic and dopaminergic neurotransmission, alleviating Ca^2+^ imbalance and decreasing excitability within the enteric nervous system [45].

**Anti-inflammatory activity** The role of KYNA in mitigating inflammation has been substantiated by numerous studies, with its effects primarily mediated through the aryl hydrocarbon receptor (AhR) signaling pathway. Under physiological conditions, KYNA activates the AhR pathway by upregulating G protein-coupled receptor 35 in intestinal epithelial cells. This, in turn, promotes the production of the anti-inflammatory cytokine IL-22 [16]. KYNA also alleviates inflammatory responses by inhibiting monocytes and macrophages in peripheral tissues [129] (Figure 2c). When KYN activates the AhR signal, it binds to the tumor necrosis factor-stimulated gene-6 (*TSG-6*) promoter, enhancing *TSG-6* expression. This process inhibits the production of the pro-inflammatory cytokine TNF-α through feedback mechanisms, further enhancing the anti-inflammatory capacity of the host [130,131]. Additionally, KYNA contributes to immune regulation by inhibiting helper T-cells 1 and 17 while upregulating dendritic cells to produce transforming growth factor β (TGFβ). The increased production of TGFβ promotes the function of regulatory T-cells [6,52], consequently decreasing the expression of IFN-γ [53].

**Influencing cognitive function** In a passive avoidance experimental model using mice, KYNA at concentrations of 10–20 µg/µL significantly reduced avoidance latency, whereas at a concentration of 0.25 µg/µL, it significantly increased it [54]. These findings suggest that moderate to high concentrations of KYNA may impair cognitive function in the central nervous system, whereas low KYNA concentrations might enhance memory function [132]. Recent studies have indicated that high concentrations of KYNA inhibit the glycine site of the α7nAChRs, reducing glutamatergic activity. This decreased activity subsequently disrupts signal transmission within the central nervous system, causing cognitive impairment [51,133]. Additionally, clinical studies have demonstrated that in patients with schizophrenia, pro-inflammatory cytokines IL-6 and TNF-α serve as inducers, increasing the production and accumulation of KYNA in microglial cells. Excessive levels of KYNA antagonize the NMDA receptors, thereby impairing brain function and resulting in a decline in learning and memory, potentially leading to cognitive impairment [134].

### 3.6. Xanthurenic Acid (XA)

XA is predominantly found in the brain. However, its distribution is uneven and it exists in micromolar quantities, with the highest concentration in enriched regions being approximately 1 µM [135].

**Signaling** In intercellular signaling within the central nervous system, XA can bind to specific receptors in various brain regions, playing a crucial role in signal regulation. XA facilitates neural signal transmission in the brain by activating cation channels mediated by G protein-coupled receptors and increasing intracellular Ca^2+^ concentrations [56] (Figure 2a). XA also inhibits vesicular glutamate transporters, thus preventing the uptake of glutamate into synaptic vesicles [26]. This, in turn, attenuates synaptic transmission mediated by AMPA and NMDA receptors in the prefrontal cortex and hippocampus [136]. In vitro studies have also identified XA as a ligand for type 2 and 3 metabotropic glutamate receptors; the activation of these receptors mediates sensory stimuli, such as pain. Notably, XA does not interfere with NMDA receptor-mediated excitatory responses. Thus, it primarily exerts its effects by inhibiting sensory transmission via type 2 metabotropic glutamate receptors [137]. Reduced XA expression may aid in the treatment of neurological diseases by inhibiting the endogenous activation of type 2 metabotropic glutamate receptors [26]. Notably, XA exerts significant therapeutic effects in diseases such as epilepsy and schizophrenia, which are notably associated with vesicular glutamate transporter dysfunction.

**Antioxidant activity** Currently, the experimental evidence for the antioxidant effect of XA remains insufficient [138]. Significant amounts of XA are reportedly present in the gut of *Aedes aegypti* mosquitoes, where it may exert antioxidant effects by binding to heme and iron (Fe) chelators [139] (Figure 2a).

**Inducing diseases** XA can promote the expression of TDO in the estrogen receptor/progesterone receptor/human epidermal growth factor receptor 2 pathway by activating AhR. TDO can also catalyze the metabolism of XA, maintaining AhR in an active state for prolonged periods. This consequently results in increased tumor cell migration [58]. In patients with type 2 diabetes or obesity, the levels of the cofactor pyridoxal-5-phosphate are downregulated, impeding the NAD metabolic pathway. Under chronic stress or chronic mild inflammation, this can lead to the overactivation of the KYN-XA metabolic branch, resulting in excessive XA production. XA affects the production and release of insulin by binding to it, influencing the development of diabetes [140]. Therefore, dysregulation of the tryptophan-KYN-XA pathway is one of the mechanisms leading to insulin resistance. Additionally, XA is associated with intestinal metaplasia induced by *Helicobacter pylori* infection. This bacterium can activate the cyclic GMP-AMP synthase/stimulator of the interferon genes/TANK-binding kinase 1/interferon regulatory factor 3 signaling pathway. This activation promotes the phosphorylation of IFN regulatory factor 3, leading to its nuclear translocation and increased binding to the kynurenine aminotransferase II (KAT-II) promoter. This process promotes the expression of KAT-II and induces XA production. Subsequently, XA enhances the expression of caudal-related homeobox transcription factor 2 and mucin 2 in gastric epithelial cells, thereby mediating intestinal metaplasia [60] (Figure 2d).

### 3.7. Picolinic Acid (PA)

PA is a non-selective metal ion chelator and an endogenous neuroprotective agent. It exhibits antioxidant, antifungal, antiviral, immunomodulatory, and cell growth-regulatory properties [71]. In the KYN pathway, PA is synthesized via the metabolism of 3-HAA [26]. PA levels are relatively low in the brain but are relatively elevated in peripheral organs, including the liver and kidneys [141].

**Neuroprotection** PA can mitigate the neurotoxic effects induced by QA in the central nervous system, preventing neuronal damage and the activation of infiltrating macrophages in the brain. This neuroprotective effect is likely attributable to its Zn-chelating properties [70]. Reduced PA synthesis impairs the ability to bind dietary Zn, directly contributing to diseases such as pellagra-like dermatitis, hereditary acrodermatitis enteropathica, and nutritional zinc deficiency. Furthermore, Zn deficiency disrupts the activity of zinc-dependent 5-aminolevulinic acid dehydratase, resulting in the accumulation of its substrate, 5-aminolevulinic acid, and impairing porphyrin formation. This disruption leads to porphyrin metabolism disorders, which may induce or exacerbate conditions such as pellagra [12,70]. However, the research has demonstrated that the neuroprotective effect of PA is significantly concentration-dependent. In an animal study, administering a low dose (25–250 ng) of PA into the mouse brain (excluding the hippocampus and caudate nucleus) significantly reduces seizure activity. Additionally, PA administration prolongs the latency period of seizures induced by QA [142]. Conversely, the systemic administration of high doses of PA can exert toxic effects on the hippocampus, substantia nigra, and striatum [143].

**Antiviral activity** PA exhibits significant antiviral activity against enveloped viruses. PA impacts the cell membrane functions of enveloped viruses by primarily compromising the integrity of the viral membrane, inhibiting fusion, and interfering with cellular endocytosis [98]. It also enhances antiviral efficacy by modulating immune responses. For example, in the brain tissues of patients with HIV-1 encephalitis, PA induces microglia and astrocytes to increase the expression of macrophage inflammatory protein 1 α/1 β, promoting the helper T-cell immune response [124,144] (Figure 2i).

**Antitumor effects** The synergistic effect of PA and IFN-γ in macrophage activation can significantly inhibit tumor cell proliferation. In mouse peritoneal macrophages, treatment with IFN-γ alone does not activate macrophages, whereas full activation occurs in the presence of both PA and IFN-γ. These findings suggest that PA is an effective activator of macrophages. Additionally, PA inhibits the synthesis of total RNA and the accumulation of newly synthesized 28S rRNA in macrophages, thereby stabilizing rRNA precursor levels. These regulated RNAs influence the expression of tumoricidal agents [145]. Moreover, PA can arrest cells in the G1 phase through its interaction with NAD, significantly inhibiting the continuous proliferation of tumor cells [145] (Figure 2i).

**Antifungal activity** PA enhances antimicrobial activity against *Mycobacterium avium* by restoring the expression of the antimicrobial effector molecule natural resistance-associated macrophage protein 1 in macrophages [72]. PA also potentiates the effects of antibiotics, such as clarithromycin, rifampicin, fluoroquinolones, and levofloxacin, demonstrating significant antimicrobial efficacy both extracellularly and within macrophages [72] (Figure 2i). Additionally, in vitro experiments have demonstrated that the synergistic interaction of PA and IFN-γ enhances the resistance of mouse peritoneal neutrophils to *Candida albicans* [73].

### 3.8. Cinnabarinic Acid (CA)

In the KYN metabolic pathway, CA is metabolized using 3-HAA as a substrate [146]. Under normal physiological conditions, CA is unstable and readily degrades in the presence of reducing agents, resulting in its extremely low concentrations in the cerebral cortex [147].

**Anti-inflammatory activity** The concentration of CA is approximately one-tenth that of KYN. Low doses of CA reduce the hyperactivity of pyramidal neurons and inhibit neuroinflammation, alleviating symptoms of mental disorders [148]. CA exhibits significant anti-inflammatory activity by activating the metabotropic glutamate receptor 4 and AhR [63,64]. In alcoholic liver disease, CA exhibits protective effects against alcohol-induced hepatocyte apoptosis, steatosis, and liver injury through a mechanism dependent on the AhR signaling pathway [67]. Furthermore, CA provides cellular protection by activating the stanniocalcin 2 signaling pathway, which regulates various physiological processes. These include metabolism, inflammation, oxidative stress, calcium regulation, cell proliferation, and apoptosis [65] (Figure 2f). For example, CA activation of the stanniocalcin 2 signaling pathway reduces acinar cell apoptosis and pancreatic injury [149]. Additionally, CA protects the host through various metabolic pathways. For instance, CA activates phosphorylated protein kinase B and phosphorylated extracellular signal-regulated kinase 1/2, inducing the cell cycle and preventing hydrogen peroxide-induced cell death [150]. Moreover, CA activates the signal transducer and activator of transcription 3 signaling pathway to alleviate hepatic steatosis [151].

**Regulation of glucose and lipid metabolism** In human metabolism, CA functions as a regulator of both glucose and lipid metabolism. The primary mechanism involves CA regulating specific adipocyte proteins, the endoplasmic reticulum, mitochondria, transport vesicles, and metabotropic glutamate receptor 4. This regulation enhances cellular respiration and lipid metabolism by increasing adipocyte sensitivity to hormones and neurotransmitters [66]. Additionally, CA protects the endoplasmic reticulum and lipid metabolism by reducing oxidative stress and inflammation resulting from circadian rhythm disturbances [64,65,66]. It also facilitates the interaction between blood circulation and adipose tissue by modulating gut microbiota and their metabolites, thereby ameliorating obesity in the host [66,151].

**Promoting the induction of thymocyte apoptosis** Experimental studies in animal cells have demonstrated that the generation of CA is essential for 3-HAA-induced apoptosis in thymocytes, with both compounds playing crucial roles in this process. Notably, the apoptotic-inducing activity of CA surpasses that of 3-HAA by more than tenfold [68]. The induction of thymocyte apoptosis is influenced by multiple factors. First, the oxidative conversion of 3-HAA to CA enhances CA production. The activation of cysteine-aspartic protease is critical in the induction of thymocyte apoptosis. Subsequently, CA strongly activates caspase-3. Consequently, under the influence of ROS, CA accelerates caspase-3 activation, resulting in the downregulation of mitochondrial membrane potential, and, ultimately, inducing thymocyte apoptosis [68].

### 3.9. Nicotinamide Adenine Dinucleotide (NAD)

In tryptophan metabolism, NAD is produced via the KYN pathway, a de novo synthesis route. NAD can be reduced to nicotinamide adenine dinucleotide or phosphorylated to nicotinamide adenine dinucleotide phosphate under the actions of dehydrogenases and NAD kinase, respectively [152,153]. NAD functions as a cofactor that maintains cellular redox homeostasis [76] and mediates various biological processes related to aging and disease development. These processes include redox reactions, biosynthesis metabolism, DNA repair, chromatin remodeling, cell senescence, and immune response, all of which depend on NAD [76,77,83,154].

**Maintenance of cellular redox homeostasis** In living organisms, maintaining the balance between NAD biosynthesis and degradation is essential for cellular homeostasis [98]. NAD depletion can result in mitochondrial dysfunction, leading to reduced ATP production and subsequent increases in ROS production and oxidative stress. This cascade leads to DNA damage and overactivation of DNA repair enzymes. This further depletes NAD-dependent deacetylases, triggers cell death, and ultimately contributes to aging [155,156]. Mitochondrial dysfunction resulting from disruptions in NAD homeostasis can promote insulin resistance or lipid metabolism disorders. This effect is mediated via the silent information regulator 1 and deacetylase sirtuin 3 signaling pathways. These disruptions contribute to the onset of metabolic disorders, including obesity, diabetes, hepatic steatosis, non-alcoholic fatty liver disease, and kidney diseases [157].

Retinal ganglion cell axons and dendrites depend on NAD for energy production and maintaining cellular functions. Impaired NAD synthesis can cause mitochondrial dysfunction in these neurons, eventually culminating in neuronal degeneration [158]. In the hypothalamus, NAD is significantly associated with functions such as circadian rhythm regulation [159], directly impacting mitochondrial and metabolic functions [160]. Additionally, it can influence hormone synthesis and secretion to regulate neuroendocrine pathways [161]. Alterations in NAD homeostasis in the hypothalamus can disrupt NF-κB signaling, induce neurotoxicity, exacerbate microglial activation, and promote hypothalamic inflammation. This, in turn, accelerates aging and contributes to obesity [161,162].

**Maintenance of immune cell function** NAD is closely associated with liver health, promoting liver regeneration and protecting it from hepatotoxicity [163]. NAD also provides cardiovascular protection by reducing immune cell infiltration and matrix degradation. Consequently, the formation of abdominal aortic aneurysms is decreased, providing vascular protection [164]. NAD regulates the depolarizing sodium current (*INa*) and cardiac electrical activity in heart sodium channels, exerting anti-arrhythmic effects [164,165]. Maintaining the balance of NAD/NADP can alleviate myocardial necrosis, fibrosis, and inflammatory markers, improve myocardial metabolism and antioxidant capacity, and promote the recovery of cardiac function [164]. Moreover, muscle function gradually declines with age. Notably, increasing NAD levels not only significantly improves muscle function and type but also enhances oxidative metabolism within the muscles. This leads to enhanced endurance and thermogenic capacity [82,163]. Furthermore, a previous study indicated a significant increase in NAD levels in the serum of individuals with intestinal inflammation. This increase is closely associated with the expression and enzyme activity of poly polymerase 1, (NAD+)-dependent sirtuin deacetylases, and ADP-ribosyl cyclase in the intestinal mucosa. It also promotes the release of inflammatory cytokines such as IL-1β, IL-6, and TNF-α [166].

Importantly, restoring NAD levels has emerged as a promising strategy for treating various conditions. It has been demonstrated to be effective in restoring cytotoxic T lymphocyte activity [167], improving cognitive function [77], enhancing neural stem cell proliferation and regeneration capacity [168], and alleviating intestinal diseases such as mesenteric ischemia and colitis [168].

### 3.10. Nicotinic Acid (NA)

NA, an intermediate metabolite in the KYN pathway, also serves as a dietary component of vitamin B3. NA functions as a metabolic precursor of NAD [86]. However, it has garnered less attention compared to other metabolites in the pathway, such as KYN, KYNA, and QA. Dietary NA is significantly less efficient than tryptophan in replenishing NAD and exerts limited effects on peripheral tissues [169]. Excessive supplementation of NA may result in elevated levels of N1-methyl-2-pyridone-5-carboxamide and N1-methyl-4-pyridone-3-carboxamide, thereby increasing the risk of cardiovascular disease [83].

**Regulating lipid metabolism** NA plays a crucial role in lipid metabolism regulation, primarily by reducing blood lipids, improving lipoprotein profiles, and modulating inflammation in adipose tissue. Research has indicated that NA reduces the degradation of high-density lipoprotein–apolipoprotein (apo) AI complexes and inhibits triglyceride synthesis by downregulating the expression of the β-chain ATP synthase on hepatocyte surfaces [74,87]. Furthermore, NA directly targets vascular endothelial cells, suppressing vascular oxidation and inflammatory responses. It also decreases ROS production and downregulates the expression of vascular cell adhesion molecule-1 and monocyte chemoattractant protein-1 genes [87]. The role of NA in lipid metabolism regulation offers a significant theoretical foundation for treating dyslipidemia and atherosclerosis. However, during lipid metabolism regulation, NA can enter cells through transporter-mediated pathways and activate transient receptor potential vanilloid 1, resulting in skin vasodilation [88]. Prolonged intake of high-dose NA may induce methyl depletion, oxidative stress, and insulin resistance, exacerbating the progression of type 2 diabetes [89]. Thus, developing novel lipid regulation strategies leveraging the functions of NA remains a significant challenge.

### 3.11. Anthranilic Acid (AA)

In the KYN pathway, AA is an intermediate metabolite and one of the precursors of 3-HAA [90]. Similar to KYN and 3-HK, AA can effectively cross the blood–brain barrier [70].

**Antioxidant activity** AA is an effective chelator of metal ions. In organisms, AA can form complexes with Fe^2+^, downregulating ROS levels, and exhibiting antioxidant activity [70] (Figure 2a).

**Promoting the development of neurodegenerative diseases** Animal experiments have indicated that AA inhibits the citric acid cycle and respiratory chain complexes I-III, disrupting mitochondrial function. This disruption may mediate the development of neurodegenerative diseases such as Parkinson’s, Huntington’s, and Alzheimer’s diseases [84].

### 3.12. N′-Formyl Kynurenine (NFK)

The oxidation of tryptophan to NFK is the first step in the KYN pathway, catalyzed by TDO or IDO [90].

**Inducing impairments in learning and cognitive functions** Currently, research on NFK remains limited. NFK is primarily associated with cognitive impairment or working memory deficits. High concentrations of NFK may mediate oxidative capacity damage, leading to impaired working memory function. However, the underlying mechanisms are still under investigation [170].

## 4. Physiological Activity and Mechanism of Action of Kynurenine Pathway Metabolites in Diseases

The metabolites of the KYN pathway play dual roles in neuroprotection and neurotoxicity, serving distinct functions in both physiological and pathological processes. Maintaining homeostasis of the KYN pathway is crucial for preventing and treating various diseases.

### 4.1. The Kynurenine Pathway and Neurodevelopmental Disorders

**Attention deficit and hyperactivity disorder (ADHD)** ADHD is a neurodevelopmental disorder characterized by deficits in sustained attention, hyperactivity, and emotional impulsivity [171]. Research has demonstrated a significant association between the KYN metabolic pathway and ADHD. Compared to healthy individuals, patients with ADHD exhibit elevated blood levels of KYN, with significantly reduced levels of KYNA and 3-HK. This suggests that an imbalance in the metabolite levels within the KYN metabolic pathway may be associated with the pathogenesis of ADHD [172,173,174]. This metabolic characteristic is also observed in patients with depression and bipolar disorder [175]. Analytical mechanistic analyses have indicated that a reduction in KYNA levels diminishes its regulatory influence over the competitive inhibition of ionotropic glutamate and NMDA receptors. This, in turn, leads to the loss of its antagonistic effect on QA toxicity [51,176,177], diminishing the neuroprotective effect of KYN. ADHD-related fatigue is also mediated by the abnormal enhancement of the tryptophan–KYN metabolic pathway. Tryptophan influences the recovery of attention and cognitive functions impaired by central fatigue by competing with neutral amino acids, including branched-chain amino acids, for absorption. Thus, regulating the balance of the KYN pathway is a potential strategy to alleviate ADHD symptoms and related fatigue issues [178]. Administering methylphenidate to children with ADHD can restore the homeostatic balance of the KYN metabolic pathway, increase KYNA levels, and decrease QA levels, alleviating multiple clinical symptoms [179]. Therefore, monitoring changes in the KYN metabolites as inflammatory markers could help track the progression and severity of ADHD, guiding subsequent treatment plans.

**Autism spectrum disorder (ASD)** ASD is a complex neurodevelopmental disorder, characterized by qualitative impairments in social interaction, communication abnormalities, and repetitive stereotyped behaviors [180]. Although the pathogenesis of ASD is intricate and challenging to elucidate, the KYN metabolic pathway is suggested to play a pivotal role in its development. However, conflicting conclusions have been reported in ASD-related studies. For example, a previous study has indicated significantly reduced serum 3-HAA levels but increased 3-HK and KYNA levels in individuals with ASD compared to those of neurotypical individuals [181]. One study has also proposed a link between the onset of ASD and reduced KYNA levels, which are associated with heightened glutamatergic excitotoxicity [181]. Conversely, other studies have found no significant differences in KYNA levels between individuals with ASD and neurotypical individuals [182]. Although excessively high KYNA levels may impair cognitive function, KYNA also scavenges free radicals and alleviates oxidative stress-induced mitochondrial dysfunction [177]. Some researchers have posited that reduced KYNA levels may contribute to ASD pathogenesis. For instance, simulation experiments using *Ptchd1*-knockout mouse models of ASD revealed reduced KYNA levels in both the serum and brain. Notably, increasing KYNA levels in these mice reportedly reverses several behavioral abnormalities [183]. These findings suggest that the KYN metabolic pathway is implicated in ASD development, with KYNA playing a potentially critical role. In addition to KYNA, alterations in KYN levels under inflammatory conditions are strongly associated with ASD development [95,184]. Inflammatory responses activate various cells, including microglia and macrophages, thereby enhancing IDO activity, which increases KYN levels and consumes NAD [185]. This process may elevate QA levels, affecting neuroimmune [186] and synaptic functions [177] through enhanced glutamatergic neurotransmission. Such alterations may impact ASD development. Finally, as the KYN pathway is the primary route for tryptophan metabolism, excessive activation under inflammatory conditions may suppress the 5-HT metabolic pathway. This, in turn, decreases the levels of melatonin and 5-HT, both of which exhibit restorative effects on social deficits and sleep disturbances in ASD [187,188]. These findings suggest that broader alterations in the KYN metabolic pathway may also contribute to ASD pathogenesis. However, factors such as age, sex, and immune status significantly influence ASD-related findings. These factors, along with broader challenges in ASD mechanism research, contribute to its complexity, warranting additional research.

### 4.2. The Kynurenine Pathway and Mental Illness

**Depression** Decreased levels of 5-HT, an essential neurotransmitter, are closely associated with depressive symptoms [6,189]. A study has indicated that in patients with depression, the KYN pathway is dysregulated, with increased IDO activity, leading to more tryptophan being metabolized through the KYN pathway [190]. Given that tryptophan is a precursor for synthesizing 5-HT, its extensive metabolism through the KYN pathway results in reduced 5-HT synthesis [51,191]. Additionally, QA is neurotoxic and can activate NMDA receptors, causing excitotoxicity and neuronal damage [192,193]. This neurotoxicity may contribute to the abnormal brain function observed in patients with depression.

**Schizophrenia** Glutamate is the principal excitatory neurotransmitter in the brain, and its dysfunction is linked to symptoms such as hallucinations and delusions in schizophrenia [194]. A previous study has demonstrated that the levels of KYN and KYNA in the cerebrospinal fluid of individuals with schizophrenia are altered [195]. These abnormalities in KYNA levels may disrupt the glutamatergic system, impairing cognitive, emotional, and other brain functions [196,197].

### 4.3. The Kynurenine Pathway and Neurodegenerative Diseases

**Alzheimer’s disease (AD)** AD is among the most prevalent forms of dementia in older adults. In AD, the primary pathological features include Aβ accumulation, tau protein phosphorylation, synaptic transmission dysfunction, and neuroinflammation [196]. In patients with AD, the tryptophan–QA pathway metabolism is enhanced, resulting in increased QA accumulation, which exacerbates AD symptoms through neurotoxic effects [198]. Moreover, QA immunoreactivity is reportedly elevated in the microglia and astrocytes of the hippocampus of patients with AD [197]. QA activates NMDA receptors, enhancing tau protein phosphorylation [199]. It also regulates glutamate release and inhibits its uptake by cells, causing excitotoxicity [200]. Conversely, QA induces astrocytes or neurons to produce ROS, damaging intracellular antioxidant defense systems, and leading to oxidative stress and mitochondrial dysfunction. This results in synaptic dysfunction and neuronal death, exacerbating the pathological progression of AD [200]. Additionally, QA can stimulate astrocytes to produce monocyte chemoattractant protein-1 and IL-1, leading to neuroinflammation [197]. The inflammatory response can lead to tau protein phosphorylation, which subsequently promotes the production of immune factors, such as IL-6, and ROS, by neuronal cells. This production results in neuronal and axonal damage, further promoting Aβ accumulation [201,202,203].

In addition to QA, 3-HK has also been implicated in the progression of AD. 3-HK induces oxidative stress in neuronal cells, impairing mitochondrial function. This, in turn, exacerbates the degeneration of memory and motor control regions in the brain, thereby accelerating AD progression [198]. Furthermore, research has demonstrated that 3-HK and 3-HAA, acting as metal chelators, enhance their toxicity to mitochondrial and cellular functions by chelating Cu^2+^ in primary astrocyte cultures [199]. This process significantly damages brain tissue and promotes Aβ accumulation.

However, in response to the toxic effects of QA and 3-HK, astrocytes produce a significant amount of KYNA. Notably, KYNA mitigates intracellular Ca^2+^ overload, thereby preserving mitochondrial function [200,204]. Furthermore, KYNA acts as a scavenger of ROS, demonstrating antioxidant properties and alleviating oxidative stress [200]. These physiological mechanisms possess significant potential to prevent the onset of AD. During AD progression, multiple metabolites of the KYN pathway contribute to the disease, with the pathological process being driven by their combined regulatory effects.

**Parkinson’s disease (PD)** PD primarily arises from the degeneration and death of dopaminergic neurons in the substantia nigra of the midbrain. PD is mainly characterized by the dysfunction of dopaminergic neurons and resultant motor impairments [205,206]. Patients with PD exhibit elevated IDO expression and activation of the KYN pathway in the substantia nigra. Abnormal changes in KYN pathway metabolites, driven by aging and inflammation, are associated with an increased risk of PD onset [207,208]. Studies have reported reduced KYNA levels in patients with PD [209], while QA levels are elevated in the striatum and cortex [210]. Moreover, 3-HK, interacts with dopamine, generating oxidative stress products that damage dopaminergic neurons. Numerous studies have demonstrated that increasing KYNA levels and reducing QA levels mitigate neurotoxicity, oxidative stress, and inflammation, thereby improving PD progression [18,211]. Although the primary mechanisms underlying the pathology of PD remain unclear, alterations in KYN pathway metabolite levels may serve as biomarkers to monitor disease progression and offer diagnostic insights into treatment.

### 4.4. The Kynurenine Pathway and Cardiovascular Diseases

**Atherosclerosis (AS)** In AS, the inflammatory response plays a pivotal role in the onset of the disease. The KYN pathway is intricately associated with the inflammatory process within the vessel wall [212,213]. Upon endothelial cell damage, IDO is activated, triggering the initiation of the KYN pathway [213]. Metabolites such as QA produced via the KYN pathway facilitate monocyte adhesion to endothelial cells. This is followed by their migration into the subendothelial space, differentiation into macrophages, and lipid uptake, ultimately resulting in foam cell formation [213]. The accumulation of foam cells represents an early indicator of atherosclerotic plaque formation [214]. Concurrently, the release of inflammatory cytokines further stimulates the proliferation and migration of smooth muscle cells, contributing to vessel wall thickening and exacerbating AS progression [215].

### 4.5. The Kynurenine Pathway and Autoimmune Diseases

**Rheumatoid arthritis (RA)** IDO expression is elevated in the synovial tissue of patients with RA [85,216]. This increased expression triggers activation of the local KYN pathway, leading to elevated levels of KYN and its metabolites, which subsequently impact the function of immune cells, including helper T-cells [85,217]. Furthermore, the activation of the KYN pathway stimulates the production of local inflammatory cytokines [216,218], such as TNF-α and IL-6, exacerbating joint inflammation and damage.

**Inflammatory bowel disease (IBD)** In the intestinal tissues of patients with IBD, abnormal expression of IDO is observed. This abnormal expression results in an increase in KYN pathway metabolites, compromising the integrity of the intestinal mucosal barrier [219]. QA increases intestinal permeability, facilitating the entry of intestinal antigens into the body. This triggers immune responses and contributes to the persistence and exacerbation of intestinal inflammation [220].

### 4.6. The Kynurenine Pathway and Tumors

Within the tumor microenvironment, tumor cells modulate the KYN pathway through diverse mechanisms. For example, tumor cells induce the expression of IDO, resulting in tryptophan depletion in adjacent immune cells [221,222]. The depletion of tryptophan impairs T lymphocyte function, enabling tumor cells to evade immune surveillance [221,222,223]. Additionally, certain metabolites generated by the KYN pathway, such as 3-HK, induce oxidative stress. This stress damages nearby normal tissue cells and alters the physiological microenvironment to promote tumor growth, invasion, and metastasis [224,225]. For example, in tumors such as melanoma, these KYN pathway-mediated mechanisms of immune suppression and tissue damage are particularly pronounced, establishing conditions conducive to tumor progression [225].

### 4.7. The Kynurenine Pathway and Chronic Kidney Disease5

**Chronic Kidney Disease (CKD)** The kidney is a primary organ for tryptophan metabolism, and its pathological characteristics are closely associated with the KYN pathway. Abnormal immune regulation, uremic toxin production, and vascular lesions are all associated with the KYN pathway. A characteristic of CKD is the elevated expression of AhR, with KYN serving as an endogenous activator. Animal studies have demonstrated that inhibiting AhR activation by KYN reduces the fibrotic expression of fibronectin, collagen I, and α-smooth muscle action in CKD. This downregulated expression alleviates the disease [226,227]. Enzyme activity analysis has revealed significantly elevated activities of KMO and IDO in various kidney diseases, including acute kidney injury, CKD, polycystic kidney disease, and end-stage kidney disease [228]. These elevated activities are accompanied by a marked increase in the KYN/tryptophan ratio, indicative of KYN accumulation in patients [140]. As CKD progresses, QA levels in the body increase, resulting in neurological complications such as uremic encephalopathy. Another study has confirmed that KYN, KYNA, and QA are positively correlated with the severity of renal function disorders and induce the elevation of the pro-inflammatory cytokines TNF-α and IL-6 [229]. Conversely, a decrease in KYN levels can enhance the anti-inflammatory response in the host, preventing hyperuricemia-induced CKD [226]. Furthermore, research has identified elevated levels of 3-HK in secondary adrenal insufficiency, suggesting it is a biomarker for monitoring this condition [230]. Accumulation of KYN may suppress downstream metabolites such as NAD and XA. This suppression can affect insulin production and release, which can contribute to diabetes and kidney disease, potentially leading to severe complications [140,231].

### 4.8. The Kynurenine Pathway and Diabetes Mellitus

Activation of the KYN pathway generates several inflammation-related metabolites. For instance, elevated IDO activity increases KYN levels, activating inflammatory signaling pathways [6]. These inflammatory signals interfere with insulin signaling, resulting in insulin resistance [232]. In adipose tissues, the inflammatory response decreases tyrosine phosphorylation while increasing serine phosphorylation of insulin receptor substrates, thereby diminishing insulin action [233,234,235]. This process elevates blood glucose levels, exacerbating diabetes. KYN pathway metabolites also directly damage pancreatic β-cells, which are responsible for insulin secretion and are essential for blood glucose regulation [236]. Metabolites such as 3-HK induce oxidative stress responses in the body, resulting in mitochondrial dysfunction, endoplasmic reticulum stress, and other intracellular damage in pancreatic β-cells [237,238]. These damages impair the insulin secretion function of pancreatic β-cells, affecting blood glucose control [5,236]. Notably, prolonged hyperglycemia results in the aberrant activation of the KYN pathway in pancreatic β-cells, exacerbating their dysfunction [239,240].

## 5. Conclusions and Perspectives

The KYN pathway is the primary route of tryptophan metabolism, significantly impacting physiological and biochemical states and the occurrence and progression of diseases. Metabolites of the KYN pathway exhibit dual effects under varying physiological conditions. At elevated levels, KYN, QA, 3-HK, and 3-HAA accumulate in the brain, primarily exhibiting neurotoxic effects. These metabolites induce or exacerbate excitotoxicity, oxidative stress, and inflammatory responses through the central nervous system. This ultimately promotes the development of neurodegenerative and neurological diseases. In contrast, KYNA, XA, CA, and PA exhibit protective effects. At steady levels, these metabolites regulate toxic metabolites in the metabolic pathway and participate in antioxidant, anti-inflammatory, antibacterial, and antitumor functions. However, under specific physiological conditions, the functions of the KYN pathway metabolites can be reversed [241]. Moreover, the metabolites of the KYN pathway do not act independently; they coordinate or antagonize each other, forming a complex regulatory network. For example, KYNA and XA influence each other in neural signal regulation, collectively affecting brain neural activity. PA and 3-HAA closely interact in immune–inflammatory responses and apoptotic processes, finely tuning immune homeostasis and cell fate.

The physiological functions of the KYN pathway metabolites suggest potential therapeutic targets for related diseases, driving research into targeted drugs and offering new treatment strategies. Among KYN metabolites, only tryptophan, KYN, and 3-HK can cross the blood–brain barrier; hence, overcoming this barrier presents a significant challenge [70].

In addition to the KYN pathway, tryptophan is also metabolized through the 5-HT pathway and microbial metabolic pathways [242]. The KYN pathway is closely intertwined with the 5-HT pathway and microbial metabolic pathways of tryptophan. Metabolites of the 5-HT pathway regulate the neuropsychiatric system and synergize with KYN pathway metabolites to influence cognition, mood, and inflammation, thereby affecting overall physiological functions. Tryptophan metabolized by gut microbiota generates various active metabolites within microbial metabolic pathways. Among these, indole metabolites can cross the blood–brain barrier. Moreover, consuming tryptophan metabolized by gut microbiota can elevate indole metabolite levels in the brain [243]. These metabolites interact through the brain–gut axis, liver–gut axis, and KYN pathway metabolites. Together, they contribute to the development of gut and systemic diseases. This highlights the integrative and complex nature of the metabolic network of the body and provides a systemic perspective for disease research. For instance, certain indole derivatives produced by gut microbes, such as indole-3-propionic acid, prevent the aggregation of Aβ-like proteins, reduce neuroinflammation, enhance neuronal survival, and promote axon regeneration and repair [244,245,246]. Moreover, nutritional intake is pivotal in modulating the KYN metabolic pathway. Many research studies have indicated that diverse dietary patterns, along with nutrient intake or deficiency, significantly impact this pathway through mechanisms such as substrate provision, enzyme activity modulation, inflammation suppression, and oxidative stress reduction. For example, a high-fat diet could disrupt the interaction between gut microbiota and colonic cells, impairing the KYN metabolic pathway in peripheral organs through a gut microbiota-dependent process. This leads to elevated serum levels of 3-HK, KYN, and the Kyn/tryptophan ratio, thereby upregulating the kynurenine pathway and disrupting serum tryptophan metabolism [247]. Similarly, a methyl-deficient diet could elevate the plasma concentrations of KYN and its associated metabolites, including KYNA and XA [248]. Furthermore, similar to dietary patterns, nutrient intake can also significantly influence the kynurenine (KYN) pathway, such as a sufficient intake of vitamins C and E neutralizing free radicals, like reactive oxygen species induced by 3-HK, thereby safeguarding neurons from oxidative damage [121]. Polyphenols in plants, including chlorogenic acid and quercetin, could compete with KYN for the AhR, thereby mitigating inflammatory responses [249]. Short-chain fatty acids (SCFAs) could alleviate inflammation caused by metabolites in the kynurenine pathway by suppressing IDO expression [250]. In summary, nutrition exerts a multifaceted impact on the KYN pathway, whether at the metabolite level or in terms of function. Dietary patterns, nutrients, and gut microbiota could interact collectively modulate the KYN pathway activity. A comprehensive investigation of these mechanisms is essential for understanding disease pathogenesis and developing innovative therapeutic strategies.

Despite significant advances in the study of KYN pathway metabolites, numerous critical issues remain unresolved. Future research should prioritize the following areas. First, an in-depth analysis of the molecular functions and cell signaling mechanisms of metabolites. Such studies should involve advanced technologies, including gene editing, single-cell sequencing, and proteomics, to precisely identify their targets and regulatory networks in both physiological and pathological contexts. Second, a strong emphasis should be placed on clinical translational research to investigate potential biomarkers and therapeutic targets based on the association between metabolite functions and diseases, as well as developing novel, targeted drugs and precise nutritional intervention strategies. Third, the synergistic effects between metabolites and various metabolic pathways should be considered to comprehensively elucidate the dynamic changes in the tryptophan metabolic network in health and disease. This would provide new theoretical and practical support for the precise diagnosis, treatment, and management of human diseases.

## Figures and Tables

**Table 1:** KYN pathway metabolites.

Metabolites	Sites of Metabolic Activity	Metabolic Enzyme	Associated Physiological Functions
Kynurenine (KYN)	Microglia, astrocytes, macrophages, liver, kidneys, and brain [24,25,26]	Kynurenine aminotransferases ^1^ (KATs)	Neurotoxicity [27], promotion of oxidative stress [28], growth inhibition, apoptosis induction [28], and free radical-scavenging (hydroxyl radicals and peroxynitrite anions) [29,30]
3-Hydroxykynurenine (3-HK)	Macrophages, microglia, astrocytes, retina, iris/ciliary bodies, and eye lenses [26,31,32]	Kynurenine 3-monooxygenase ^2^ (KMO)	Neurotoxicity [33], antioxidant activity (low concentrations) [34], pro-oxidant activity (high concentrations) [34], cataract induction [32,35], ultraviolet light filtration [35], and cognitive function [35]
3-Hydroxyanthranilic acid (3-HAA)	Microglia and astrocytes [26]	Kynureninase ^1^ (KYNU)	Antioxidant activity [36,37], free radical scavenging [38], anti-inflammatory activity [38], neuroprotection [38], and genotoxicity (Cu^2+^ chelation) [39]
Quinolinic acid (QA)	Macrophages, microglia, dendritic cells, and other immune cells [26,40]	3-Hydroxyanthranilate 3,4-dioxygenase ^3^ (HAAO)	Neurotoxicity [41], pro-inflammatory activity [42], induction of cell death [43,44,45], oxidative stress promotion [35], influencing cognitive function [46,47,48,49], and physiological protection [50,51]
Kynurenic acid (KYNA)	Astrocytes [26]	Kynurenine aminotransferases ^1^ (KATs)	Neuroprotection [6], anti-inflammatory activity, immune modulation [16,52,53,54], and cognitive function regulation (at high concentrations) [54]
Xanthurenic acid (XA)	Brain cortex or renal cortex [26,55], eye lenses [32]	Kynurenine aminotransferases ^1^ (KATs)	Signaling [26,56], neuroprotection, antioxidant activity [57], promotion of tumor cell proliferation [58], disruption of insulin function [59], and promotion of gastrointestinal epithelial metaplasia [60]
Cinnabarinic acid (CA)	Liver [26], kidneys, spleen, and lungs [61,62]	/	Anti-inflammatory activity [63,64], calcium regulation [65], regulation of glucose and lipid metabolism, antioxidant activity [66], cellular protection [67], and antibacterial activity [68]
Picolinic acid (PA)	Liver, kidney [69]	3-Hydroxyanthranilate 3,4-dioxygenase ^3^ (HAAO)	Metal chelation [12,70], neuroprotection [70], antioxidant activity [71], antifungal activity [72,73], antiviral activity [74], immune modulation, and cell growth regulation [21]
Nicotinamide adenine dinucleotide (NAD)	Mainly in mitochondria-rich cells such as liver cells [75]	Quinolinate phosphoribosyltransferase ^4^ (QPRT)	Maintenance of cellular redox homeostasis [76], antitumor activity impairment, regulatory metabolism, DNA repair, chromatin remodeling, cellular senescence, immune cell function, and neuronal plasticity [76,77,78,79,80,81,82,83]
Anthranilic acid (AA)	Brain cortex [84]	Kynureninase ^1^ (KYNU)	Antioxidant activity [71]; it also serves as a marker of inflammation [85]
Nicotinic acid (NA)	Liver and intestine [86]	Nicotinate phosphoribosyltransferase domain-containing 1 ^5^ (NAPRT1)	Improvement of pellagra [12], lipid metabolism regulation [74,86,87], anti-inflammatory activity, antioxidant activity [87], a cause of flushing [88], oxidative stress activity, and insulin resistance [89]
*N*’-formylkynurenine (NFK)	Liver [90]	Tryptophan 2,3-dioxygenase ^4^ (TDO) or indoleamine 2,3 dioxygenase ^4^ (IDO)	Impaired working memory function (high concentrations) [89]

Note: The function of enzymes on metabolites (^1^: aminotransferase; ^2^: hydroxylase; ^3^: oxidase; ^4^: rate-limiting enzyme; ^5^: salvage enzymes).

## Data Availability

No new data were created or analyzed in this study.
